# Adaptive Weighted Factor Graph Optimized Positioning Algorithm Based on Joint GNSS/INS/Vision Residual Detection

**DOI:** 10.3390/s26123783

**Published:** 2026-06-14

**Authors:** Jin Wang, Jun Zou, Yan Xing, Jin Lu, Pengwu Wan, Jianbo Du

**Affiliations:** 1 School of Communications and Information Engineering, Xi’an University of Posts and Telecommunications, Xi’an 710121, China; 230121245@stu.xupt.edu.cn (J.Z.); lujin@xupt.edu.cn (J.L.); wpw_lz@126.com (P.W.); dujianboo@163.com (J.D.); 2 National Time Service Center, Chinese Academy of Sciences, Xi’an 710600, China; xingyan@ntsc.ac.cn

**Keywords:** GNSS/INS/vision, joint residual, adaptive weighting, factor graph optimization

## Abstract

Multi-sensor fusion of GNSS, IMU, and vision sensors has been extensively applied in urban Internet of Things systems and automated driving to improve positioning accuracy in complex environments. However, conventional FGO algorithms are based on fixed sensor weights, which limit their adaptability to fluctuations in sensor errors caused by environmental changes, thereby compromising positioning performance. To overcome this limitation, a novel multi-sensor adaptive weighted localization algorithm based on joint residuals detection was proposed in this study. The algorithm computes joint residuals by the sliding window accumulation of GNSS, IMU, and vision sensor measurements. By integrating a global weight decay factor into the M-estimation framework, the weights of each sensor were dynamically adjusted, thereby suppressing the effects of outliers on the state estimation. This approach enables high-precision and robust estimation of position, velocity, and attitude. Experimental results demonstrate that, based on validation with the GNSS–Visual–Inertial Navigation System (GVINS) public datasets sports field and complex environments, the proposed method exhibits superior performance in challenging low-altitude economic scenarios such as weak GNSS signals and significant IMU drift—specifically, it improves positioning accuracy by 32.3% and reduces velocity error by 32% compared to traditional FGO algorithms. In scenarios with GNSS signal interference, the system effectively mitigates error accumulation and maintains the stability of position and velocity estimation. The proposed algorithm demonstrates exceptional positioning accuracy and robustness in complex and dynamic environments, making it highly suitable for advanced urban IoT and automated driving applications.

## 1. Introduction

In modern navigation and positioning systems, obtaining high-precision positional information is essential for applications such as autonomous driving, smart cities, and unmanned aerial vehicles. However, conventional positioning methods, including global navigation satellite systems and INS, have significant limitations in complex environments. GNSS signals are highly susceptible to disruptions in challenging scenarios such as urban canyons, tunnels, and densely forested areas, and inertial measurement unit errors accumulate over time, resulting in a rapid decline in positioning accuracy. To overcome these challenges, GNSS are frequently integrated with inertial measurement units and vision sensors to leverage their complementary strengths and improve navigation and localization performance [1,2,3].

The Kalman filter and its extended variants are widely used to combine GNSS, IMU, and vision sensors in various navigation and localization systems. For example, Li et al. integrated GNSS, INS, and vision sensors using a Kalman filter-based framework, whereas the Rauch–Tung–Striebel smoother, also known as the forward–backward smoother, was used to achieve optimal attitude estimation [4]. Similarly, Angelino et al. described a state-estimation system that fuses GNSS solutions with visual and inertial data within an EKF framework [5]. Furthermore, Shen et al. presented a traceless Kalman filter algorithm that combines visual, inertial, LiDAR, and GNSS data to generate smooth and consistent trajectories in different environments [6]. Building on these advances, Sun et al. proposed a robust two-step integration algorithm based on an EKF. In this method, IMU, GPS, and vision sensor data are fused using incomplete constraints to improve the vehicle state estimation in diverse urban scenarios [7]. Although the Kalman filter and its variants are effective, they have inherent problems in handling nonlinear errors. These methods rely solely on the current state and do not retain information from past states, making it impossible to leverage past data for releasing or iterative optimization. Consequently, their ability to improve estimation accuracy of the current state is inherently limited [8].

To overcome these limitations and better handle nonlinear models while leveraging a batch of measurements for improved accuracy, FGO methods have gained significant traction in recent years [9,10,11]. The FGO represents multi-sensor systems as factor graph models, where the optimal state estimate is derived by computing the maximum a posteriori estimate of the joint probability distribution, which is the foundation of the approach presented in this work. Compared to the conventional Kalman filter methods, FGO can provide improved accuracy in practical state estimation when dealing with nonlinear models. This is because the batch optimization process in FGO allows for multiple iterations and relinearization of measurement models within the sliding window, which can converge to a more optimal solution compared to the single-step update in EKF, especially when initial state estimates are poor or models are highly nonlinear. For example, Ma et al. presented a multi-sensor fusion navigation framework based on the FGO, which integrates tightly coupled visual information from optical arrays [12]. This approach enables high-precision, high-frequency relative position estimation between an autonomous underwater vehicle and its moving dock during the terminal docking phase. Similarly, Mascaro et al. proposed a multi-sensor fusion method that uses decoupled graph optimization to estimate the global 6-DOF attitude of a robot in real time [13]. This method combines a generic 6-DOF visual–inertial odometry attitude with a 3-DOF global reference position for robust and efficient localization. The FGO demonstrated exceptional performance in dynamic environments. For example, Li et al. emphasizes the use of factor graphs for tightly coupled vision and IMU data fusion [14]. This approach not only achieves highly accurate position estimation, but also provides more continuous localization information under unstable GNSS signal conditions by utilizing vision data. In addition, to address the susceptibility of visual navigation systems to complex environmental conditions, Niu et al. presented a robust real-time GNSS–visual–inertial navigation system centered on inertial navigation systems [15]. By exploiting the inherent resilience of the INS to external environmental factors, this system maximizes the advantages of the INS in terms of improving navigation performance.

However, existing FGO methods are often designed based on static data processing windows or fixed optimization strategies that do not effectively address the challenges posed by data variations in real-time environments. In particular, dynamic environments lead to significant changes in the sampling frequency and noise characteristics of sensor data, resulting in a decline in localization accuracy if conventional methods cannot adapt to these changes [16].

To solve this problem, Ben et al. proposed a robust FGO method based on dynamic kernel principal component analysis [17]. This method uses a time-lag matrix to extract nonlinear and Markov features from the incremental navigation data. In addition, a performance-monitoring system was developed to detect and evaluate faults in real time to ensure higher reliability and adaptability. Similarly, Zhou et al. derived and constructed a robust factor graph based on an improved chi-square test [18]. This approach integrates a robust factor graph with an improved chi-square test to optimize the performance of the integrated navigation algorithm to ensure greater resilience and accuracy under dynamic and uncertain conditions.

The factor graph method typically relies on empirically determined noise variance matrices to assign weights to observations, a practice that aligns with the BLUE framework, which assumes fixed noise characteristics. However, in practical applications, observation information is subject to uncertainty due to environmental dynamics, leading to fluctuations in noise variance. This violates the static assumptions of BLUE, resulting in a weighting process overly dependent on initial empirical values and incapable of dynamic adjustment [19,20]. Usually, sensors with higher empirical accuracy are assigned larger weights [21]. However, even if the performance of a particular sensor degrades significantly during data fusion, its weights remain static, thereby compromising the overall fusion performance [22]. To address these challenges, An adaptive weighting strategy based on a chi-square test was presented by Sun et al. [23]. Their approach evaluates the data quality of the GNSS and visual sensors in real time and dynamically adjusts their weights accordingly. This strategy shows strong performance in complex urban environments and significantly improves positioning accuracy.

However, the chi-square test is very sensitive to assumptions and sample sizes, which may lead to insufficient statistical effectiveness if the sample size is too small or inflated owing to the amplification of minor differences with large sample sizes. Based on this, Zhou et al. proposed a robust factor graph-optimized combination navigation algorithm that incorporates an improved chi-square test [24,25]. By incorporating a down-weighting strategy from the M-estimator, the binary results of the chi-square test were refined to increase stability and reduce false positives. Despite these improvements, the down-weighting strategy in this system remains overly simplistic and limits its ability to account for more diverse and complex scenarios.

To overcome the limitations of existing sensor weighting strategies, which are overly simplistic and perform poorly in complex and dynamic environments, and to address the lack of accurate and reliable sensor quality assessment mechanisms, this an adaptive weighted factor graph-optimized positioning algorithm based on joint GNSS/IMU/vision residual detection was proposed in this study. The main contributions of this paper can be summarized as follows:

(1) To provide robust support for subsequent integrated navigation, this study proposes an adaptive weighted factor graph optimized positioning algorithm based on joint GNSS/IMU/vision residual detection. This approach uses joint residual analysis to dynamically evaluate the quality of sensor data, enabling real-time adjustments in sensor weight assignment and ensuring improved accuracy and reliability in combined navigation systems.
(2) A proportional factor model is proposed, leveraging the joint residual test statistics of observation data and M-estimation as an adaptive weighted fusion strategy for GNSS, IMU, and vision measurements. This approach effectively improves the positioning accuracy and robustness of multi-sensor integrated navigation systems, particularly in complex and dynamic environments.

The rest of the article is organized as follows: In the second part, an overview of the system and the FGO algorithm is presented. In the third section, the proposed method is described in detail. In the fourth part, simulation experiments are carried out in different scenarios to verify the effectiveness of the proposed method. Finally, the fifth part draws conclusions from this study.

## 2. Overview

### 2.1. System Overview

**Figure 1:**
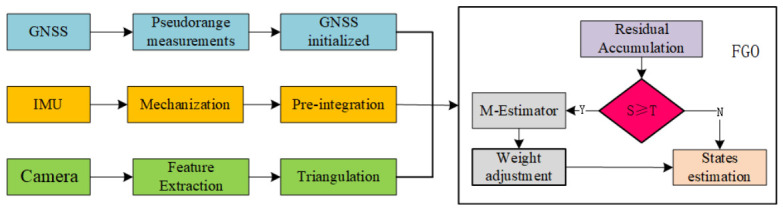
System block diagram.

A system block diagram of the proposed multi-sensor weighted fusion algorithm based on observation data residuals is illustrated in Figure 1. The algorithm consists of three primary phases. (1) The system collects preliminary position data from multiple sensors. The GNSS module receives satellite signals to generate pseudo-range measurements, the IMU module processes acceleration and angular velocity data and performs pre-integration to provide motion-related information, and the vision module extracts feature points and computes the positioning information using triangulation techniques. (2) The system evaluates the residuals of the observed data from the GNSS, IMU, and vision sensors to assess the data reliability. A test statistic is calculated and compared against a preset threshold value. If the test statistic is smaller than the threshold, the data is classified as reliable and included directly into the factor graph optimization process. If the test statistic exceeds the threshold, the data is identified as abnormal, and a down-weighting strategy is applied to minimize their influence on the fusion results. (3) Finally, the system performs factor graph optimization to fuse the multi-sensor data and generate accurate state estimation results through joint optimization, including precise position information and attitude solutions. This stepwise process ensures that the proposed algorithm effectively identifies and mitigates the effects of abnormal data, while leveraging the complementary strengths of the GNSS, IMU, and vision sensors to achieve accurate and robust positioning.

### 2.2. Frame Definition

**Figure 2:**
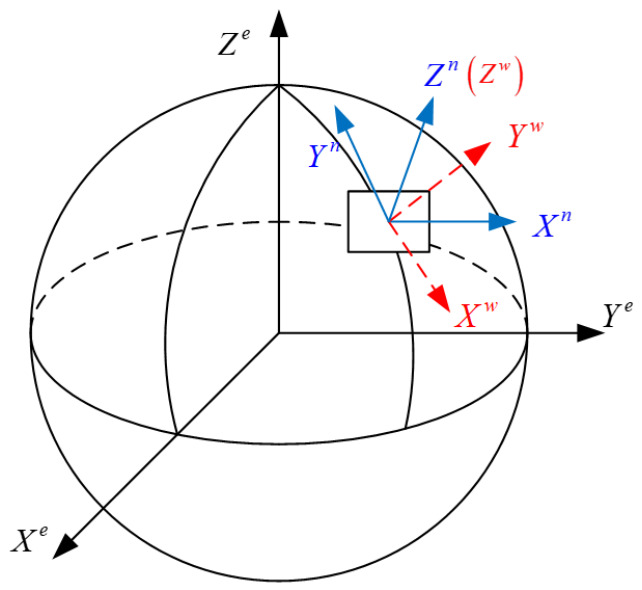
Coordinate system framework $X^{e}-Y^{e}-Z^{e}$ (geocentric geoid, e), $X^{n}-Y^{n}-Z^{n}$ (ENU coordinate system, n), $X^{w}-Y^{w}-Z^{w}$ (local world coordinate system, w).

Figure 2 illustrates the relationships between the different coordinate systems used in this study. The color scheme is defined as follows: red represents the Local World Frame ${\left(\cdot \right)}^{w}$, black represents the coordinate system framework, and blue represents the East–North–Up (ENU) frame. The specific definitions are as follows:

(1) **Sensor Frame**: The sensor coordinate system mainly includes the camera frame ${\left(\cdot \right)}^{c}$ and the IMU frame ${\left(\cdot \right)}^{i}$. In this paper, the IMU frame is chosen as the carrier body frame ${\left(\cdot \right)}^{b}$.
(2) **Local World Frame**: The visual–inertial system running frame is localized as the world frame ${\left(\cdot \right)}^{w}$, as shown in Figure 2. The origin of this frame is arbitrary, and the Z-axis is aligned with the direction of gravity.
(3) **Earth-Centered, Earth-Fixed (ECEF) Frame**: The ECEF frame is an Earth-centered fixed coordinate system whose origin is located at the Earth’s center of mass. The X-axis points to the intersection of the equator and the prime meridian. The Y-axis lies in the equatorial plane, perpendicular to the X-axis, pointing towards $90^{\circ }$ east longitude. The Z-axis points towards the North Pole along the Earth’s rotation axis.
(4) **ENU Frame**: To connect the local world coordinate system and the ECEF frame, the ENU frame is adopted in this paper. Its X, Y, and Z axes point east, north, and upward, respectively.

## 3. Methodology

In this study, a factor graph model was constructed by representing the residuals of the GNSS, IMU, and vision sensors as a factor, respectively. The best system state estimation is obtained by maximizing the a posteriori probability when all sensor measurements are known:

(1) $$\begin{array}{cc} X^{*} & =\underset{X}{\mathrm{argmax}}p\left(X|Z\right)\\ \; & =\underset{X}{\mathrm{argmax}}p\left(X\right)\prod_{i=1}^{n}p\left(Z_{i}|X\right)\\ \; & =\underset{X}{\mathrm{argmin}}\left\{{∥\varphi_{p}-H_{p}X∥}^{2}+\sum_{i=1}^{n}{∥\varphi (Z_{i},X)∥}_{P_{i}}^{2}\right\} \end{array}$$

where *X* denotes the state of the system and *Z* represents the set of n observations. $\varphi_{p}$ and $H_{p}$ are the a priori information about the system, φ(·) denotes the residual function for each measurement, and $P_{i}$ is the Mahalanobis paradigm. The core of our method lies in the dynamic adjustment of the weighting implied by $\sum_{i}$ based on real-time residual analysis.

### 3.1. GNSS Factor

The raw pseudorange measurement ${\tilde{\rho }}_{r}^{s}$ from satellite *s* to receiver *r* at epoch *k* is modeled as

(2) $$\begin{array}{cc} {\tilde{\rho }}_{r}^{s}\left(k\right) & =\rho_{r}^{s}\left(k\right)+c\left(\delta t_{r}\left(k\right)-\delta t^{s}\left(k\right)\right)+T_{r}^{s}\left(k\right)+I_{r}^{s}\left(k\right)\\ \; & +M_{r}^{s}\left(k\right)+\varepsilon_{\rho }\left(k\right) \end{array}$$

where $\rho_{r}^{s}\left(k\right)=∥P_{r}\left(k\right)-P^{s}\left(k\right)∥$ is the geometric distance between the satellite and the receiver; *c* is the speed of light in vacuum ($c\approx 3\times 10^{8}$ m/s); $\delta t_{r}$ and $\delta t^{s}$ represent the receiver clock difference and the satellite clock difference, respectively; $T_{r}^{s}$ and $I_{r}^{s}$ represent the tropospheric and ionospheric delays, respectively; $M_{r}^{s}$ is the delay caused by the multipath effect, and $\varepsilon_{\rho }$ represents the noise. Therefore, the pseudo-range residual can be expressed as

(3) $$\varphi_{\rho }=∥P_{s_{j}}-P_{r_{k}}∥+c\left(\delta t_{k}-\delta t_{s_{j}}\right)+T_{r_{k}}^{s_{j}}+I_{r_{k}}^{s_{j}}-{\tilde{\rho }}_{r_{k}}^{s_{j}}$$

where $s_{j}$ denotes the *Jth* satellite and *k* denotes the moment.

### 3.2. Inertia Factor

Inertial measurements are modeled as follows [1]:

(4) $$\begin{array}{cc} {\tilde{a}}_{t} & =a_{t}+b_{a_{t}}+n_{a}\\ {\tilde{\omega }}_{t} & =\omega_{t}+b_{\omega_{t}}+n_{\omega } \end{array}$$

where $\left\{{\tilde{a}}_{t},{\tilde{\omega }}_{t}\right\}$ is the output of the IMU at time t, $\left\{b_{a_{t}},b_{\omega_{t}}\right\}$ is the zero bias of the accelerometer and gyroscope, and *n* is the noise. Then the relative position, velocity and rotation information $\left\{{\hat{\alpha }}_{b_{t_{k+1}}}^{b_{t_{k}}},{\hat{\beta }}_{b_{t_{k+1}}}^{b_{t_{k}}},{\hat{\gamma }}_{b_{t_{k+1}}}^{b_{t_{k}}}\right\}$ from the $t_{k}$ moment to the $t_{k+1}$ moment can be obtained by pre-integrating the IMU. Therefore, the measurement residuals of the IMU can be expressed as follows [1]:

(5) $$\begin{array}{cc} \varphi_{I} & =\left[\begin{array}{c} \delta \alpha_{b_{t_{k+1}}}^{b_{t_{k}}}\\ \delta \beta_{b_{t_{k+1}}}^{b_{t_{k}}}\\ \delta \theta_{b_{t_{k+1}}}^{b_{t_{k}}}\\ \delta b_{a}\\ \delta b_{g} \end{array}\right]\\ \; & =\left[\begin{array}{c} \left(p_{b_{t_{k+1}}}-p_{b_{t_{k}}}+\frac{1}{2}g\Delta t_{k}^{2}-v_{b_{k}}\Delta t_{k}\right)-{\hat{\alpha }}_{b_{t_{k+1}}}^{b_{t_{k}}}\\ \left(v_{b_{t_{k+1}}}+g\Delta t_{k}-v_{b_{t_{k}}}\right)-{\hat{\beta }}_{b_{t_{k+1}}}^{b_{t_{k}}}\\ 2{\left[q_{b_{t_{k}}}^{-1}⊗q_{b_{t_{k+1}}}⊗{\left({\hat{\gamma }}_{b_{t_{k+1}}}^{b_{t_{k}}}\right)}^{-1}\right]}_{xyz}\\ b_{ab_{t_{k+1}}}-b_{ab_{t_{k}}}\\ b_{\omega b_{t_{k+1}}}-b_{\omega b_{t_{k}}} \end{array}\right] \end{array}$$

where $\delta \theta_{b_{t_{k+1}}}^{b_{t_{k}}}$ is the rotation error, *v* is the velocity, *p* is the position, *q* is the attitude, and $b_{t_{k}}$ is the carrier frame at the moment $t_{k}$, ${\left[\cdot \right]}_{xyz}$ returns the imaginary part of the quaternion.

### 3.3. Visual Factor

The visual projection process is modeled as follows:

(6) $$\tilde{P}=\pi_{c}\left(R_{b}^{c}\left(R_{w}^{b}X^{w}+p_{w}^{b}\right)+p_{b}^{c}\right)+n_{c}$$

where $\tilde{P}$ is the coordinate of the feature point in the image plane and $X^{w}$ is the corresponding 3D coordinate in the local world coordinate system. $\pi_{c}\left(\cdot \right)$ is the projection function of the camera, and $n_{c}$ is the measurement noise, $R_{b}^{c}$ is the rotation matrix from the body frame to the camera frame, and $R_{w}^{b}$ is the rotation matrix from the world frame to the body frame. Therefore, if a feature *l* with an inverse depth ρl in frame *i* is observed again in frame *j*, the residual difference between the two corresponding frames can be expressed as

(7) $$\varphi_{c}={\tilde{P}}_{c_{t_{j}}}-\pi_{c}\left({\hat{X}}_{c_{t_{j}}}\right)$$

(8) $$\begin{array}{cc} {\hat{X}}_{c_{t_{j}}} & =R_{b}^{c}\left(R_{w}^{b_{t_{j}}}\left(R_{b_{t_{i}}}^{w}\left(R_{c}^{b}\frac{1}{\rho l}\pi_{c}^{-1}\left({\tilde{P}}_{l}^{c_{t_{j}}}\right)+p_{c}^{b}\right)+p_{b_{t_{i}}}^{w}\right)\right.\\ \; & \;\left.+p_{w}^{b_{t_{j}}}\right)+p_{b}^{c} \end{array}$$

where $\left\{R_{c}^{b},t_{c}^{b}\right\}$ is the transformation matrix between the IMU and the camera coordinate system.

### 3.4. Sliding Window-Based Multi-Sensor Residual Accumulation with Adaptive Weighting Algorithm

The core innovation of our work is a two-layer adaptive weighting mechanism that combines global sensor reliability assessment with local observation robustness. In this system, a sliding window W is used to store the multi-sensor residuals for each epoch k, which includes the GNSS pseudo-range, IMU pre-integration, and position measurements from the vision sensors. When the sliding window receives the residuals from each sensor, they are processed sequentially and stored into the residual vector using a calendar element until the end of the sliding window. The following equation describes the process of accumulating multi-sensor residuals:

(9) $$r_{k}=\left[r_{\rho }^{\left(k\right)},r_{IMU}^{\left(k\right)},r_{vision}^{\left(k\right)}\right]$$

where $r_{k}$ is the multisensor residual vector for the kth calendar element. $r_{\rho }^{\left(k\right)}$ is a GNSS pseudo-range residual that provides the geometric constraints between the receiver and the satellite. $r_{IMU}^{\left(k\right)}$ represents the IMU pre-integrated residuals used to estimate the position, velocity, and attitude changes between neighboring calendar elements. $r_{vision}^{\left(k\right)}$ is a visual sensor position residual that provides the relative position constraints between neighboring keyframes. The sliding window helps to smooth short-term errors and provides more stable state estimates for the system by accumulating residuals from multiple calendar elements.

(1) GNSS Pseudorange Residuals

The GNSS pseudo-range measurement was realized by calculating the geometric distance between the receiver and the satellite. The pseudorange residual $r_{\rho ,i}^{\left(k\right)}$ in the *k*th calendar element represents the difference between the measured and theoretical values and is calculated as follows:

(10) $$r_{\rho ,i}^{\left(k\right)}=\frac{{\hat{\rho }}_{i}^{\left(k\right)}-\left(∥P_{r}^{\left(k\right)}-P_{s}^{\left(k\right)}∥+c\left(\delta t_{r}^{\left(k\right)}-\delta t_{s}^{\left(k\right)}\right)+T+I\right)}{\sigma_{\rho ,i}^{\left(k\right)}}$$

where ${\hat{\rho }}_{i}^{\left(k\right)}$ is the pseudorange measurement of the first satellite, $∥P_{r}^{\left(k\right)}-P_{s}^{\left(k\right)}∥$ is the geometric distance between the receiver and the satellite, and $\sigma_{\rho ,i}^{\left(k\right)}$ is the standard deviation of the pseudorange measurement. *T* and *I* represent the estimated tropospheric delay and ionospheric delay, respectively.

The pseudo-range residuals provide important geometric constraints in the optimization process, and by combining multiple satellite signals, the system can compute more accurate position information. However, GNSS signals are susceptible to multipath effects and signal occlusions, and therefore need to be combined with other sensor data to increase their robustness.

(2) IMU Residuals

The IMU sensor measurements include acceleration and angular velocity, which can be used to estimate position, velocity, and attitude changes between neighboring calendar elements. To ensure that the data from different sensors have the same scale, the IMU residuals must be normalized. The specific calculation process is as follows.

The position residuals are used to represent the difference between the position changes between the neighboring calendar elements k and j and the IMU measurements, and the normalization process can be described as follows:

(11) $$r_{pos,i,j}^{\left(k\right)}=\frac{P_{j}-\left(P_{i}+v_{i}\Delta t+\frac{1}{2}R_{i}a_{i}\Delta t^{2}\right)}{\sigma_{pos,i,j}^{\left(k\right)}}$$

where $P_{i}$ and $P_{j}$ are the positions of the calendar elements *i* and *j*, respectively, $v_{i}$ is the velocity at the calendar element *i*, $R_{i}$ is the rotation matrix from the body frame ${\left(\cdot \right)}^{b}$ to the world frame ${\left(\cdot \right)}^{w}$, $a_{i}$ is the acceleration measurement, and $\sigma_{pos,i,j}^{\left(k\right)}$ is the standard deviation of the residuals of the IMU positions.

The velocity residual of the IMU represents the deviation in the velocity change between the two calendar elements and is normalized as follows:

(12) $$r_{v,i,j}^{\left(k\right)}=\frac{v_{j}-\left(v_{i}+R_{i}a_{i}\Delta t\right)}{\sigma_{v,i,j}^{\left(k\right)}}$$

where $v_{i}$ and $v_{j}$ denote the velocities of the calendar element *i* and *j*, respectively; $\sigma_{v,i,j}^{\left(k\right)}$ is the standard deviation of the velocity residuals.

The attitude residual represents the difference between the change in attitude between neighboring calendar elements and the IMU measurements. The normalization formula for the attitude residual is as follows:

(13) $$r_{att,i,j}^{\left(k\right)}=\frac{\Delta q_{ij}-\Delta q_{IMU,ij}}{\sigma_{att,i,j}^{\left(k\right)}}$$

where $\Delta q_{ij}$ is the quaternion of attitude change and $\sigma_{att,i,j}^{\left(k\right)}$ is the standard deviation of the attitude residuals. Through this normalization process, each residual quantity of the IMU is evaluated on the same scale, ensuring that the data from different sensors can be compared fairly.

(3) Vision Sensor Residuals

The visual sensor tracks feature points in the image and calculates the relative positions between neighboring keyframes. Consistent with the tight coupling methodology described in Section 3.3, the visual residuals are defined as the reprojection error. Instead of using loose-coupled relative poses, we utilize the geometric constraint between the 3D map points and their 2D projections on the image plane. The visual residual for a feature *l* observed in frame *j* is calculated as

(14) $$r_{\mathrm{vision},l,j}^{\left(k\right)}={\tilde{P}}_{l,j}^{\left(k\right)}-\pi_{c}\left({\hat{X}}_{c_{t_{j}}}\right)$$

where ${\tilde{P}}_{l,j}^{\left(k\right)}$ is the observed 2D coordinate of feature *l* in frame *j*, and ${\hat{X}}_{c_{t_{j}}}$ is the predicted projection derived from the state estimate *X* and the camera model (Equation (8)). This formulation ensures that the visual constraints are directly integrated into the factor graph, allowing the optimization to jointly refine the camera poses and landmark positions. The residuals are normalized as follows:

(15) $$r_{\mathrm{vision},l,j}^{\left(k\right)}=\frac{{\tilde{P}}_{l,j}^{\left(k\right)}-\pi_{c}\left({\hat{X}}_{c_{t_{j}}}\right)}{\sigma_{\mathrm{vision},l,j}^{\left(k\right)}}$$

(4) Calculation of Test Statistics Within a Sliding Window

Each calendar element in the sliding window can be evaluated using a test statistic to determine if the measurements are as expected. The test statistics within the sliding window are calculated as the sum of squared normalized residuals:

(16) $$\begin{array}{cc} S_{\mathrm{GNSS}} & =\sum_{i=1}^{N_{s}}{\left(\frac{r_{\rho ,i}^{\left(k\right)}-\mu_{k}}{\sigma_{k}}\right)}^{2}\\ S_{\mathrm{IMU}} & =\sum_{j=1}^{N_{\mathrm{IMU}}}{\left(\frac{r_{\mathrm{IMU},i,j}^{\left(k\right)}}{\sigma_{\mathrm{IMU}}^{\left(k\right)}}\right)}^{2}\\ S_{\mathrm{vision}} & =\sum_{m=1}^{N_{\mathrm{vision}}}{\left(\frac{r_{\mathrm{vision},l,j}^{\left(k\right)}}{\sigma_{\mathrm{vision}}^{\left(k\right)}}\right)}^{2} \end{array}$$

Since $S_{l}$ is the sum of squares of standardized residuals, it theoretically follows a Chi-square ($\chi^{2}$) distribution with degrees of freedom equal to the number of measurements $N_{l}$. However, to balance computational efficiency and robustness against non-Gaussian noise in dynamic environments, we employ an empirical heuristic threshold $T_{l}$ rather than a strict $\chi^{2}$ quantile:

(17) $$\begin{array}{cc} T_{\mathrm{GNSS}} & =\frac{\gamma }{N_{\mathrm{GNSS}}}S_{\mathrm{GNSS}};\\ T_{\mathrm{IMU}} & =\frac{\gamma }{N_{\mathrm{IMU}}}S_{\mathrm{IMU}};\\ T_{\mathrm{Vision}} & =\frac{\gamma }{N_{\mathrm{Vision}}}S_{\mathrm{Vision}}. \end{array}$$

The threshold parameter γ is empirically set to 2.0. This value has been validated through cross-validation on the GVINS dataset to effectively suppress outliers while maintaining sufficient measurement availability. We acknowledge that this threshold does not represent a rigorous 95% statistical confidence bound but serves as a practical engineering compromise. This value provides a balanced trade-off between robustness and sensitivity. When the test statistic $S_{l}$ exceeds the threshold $T_{l}$, the data is considered potentially unreliable, and a down-weighting strategy is triggered:

(18) $$\omega_{l}=\left\{\begin{array}{cc} 1, & S_{l}\leq T_{l},\\ \omega_{l,i}, & S_{l}>T_{l} \end{array}\right.$$

where *l* represents different sensors, if $S_{l}\leq T_{l}$ indicates that the observation data of the current calendar element is of good quality, the current observation data can be trusted; if $S_{l}>T_{l}$ indicates that there may be outliers in the current observation data, the system needs to dynamically adjust the weights of different sensor types to minimize the influence of the outliers on the overall state estimation. At this point, a global weight attenuation factor $\alpha_{l}$ is introduced in this paper to adjust the weights of each sensor type in the calendar element:

(19) $$\alpha_{l}=\frac{T_{l}}{S_{l}}$$

The global weight decay factor $\alpha_{l}$ was applied to the weights of each sensor, and the specific formula to update the weights is

(20) $$\begin{array}{cc} \omega_{GNSS} & \leftarrow \alpha_{l}\cdot \omega_{GNSS}^{0}\\ \omega_{IMU} & \leftarrow \alpha_{l}\cdot \omega_{IMU}^{0}\\ \omega_{vision} & \leftarrow \alpha_{l}\cdot \omega_{vision}^{0} \end{array}$$

where $\omega_{GNSS}^{0},\omega_{IMU}^{0},\omega_{Vision}^{0}$ is the initial weight and $\omega_{GNSS},\omega_{IMU},\omega_{Vision}$ is the updated weight. This adjustment ensures that all sensor weights are weakened when the test statistic is large, thereby reducing the effect of the calendar element observations on the overall state estimation. On this basis, M-estimation is introduced to further refine the residual processing within each sensor.

M-estimation is a robust estimation method that is widely used to deal with the presence of outliers in data. Unlike the conventional least-squares method, M-estimation makes the estimation more robust by choosing different loss functions to reduce the effect of outliers on the parameter estimation. The goal of M-estimation is to minimize the objective function as follows:

(21) $$\hat{\theta }=\underset{\theta }{\arg\min}\sum_{i=1}^{n}\rho \left(r_{i}\left(\theta \right)\right)$$

where $\rho \left(r\right)$ is the loss function and $r_{i}\left(\theta \right)$ is the residual function. To solve the problem of M-estimation, it is often necessary to use a weight function obtained from the derivative $\phi \left(r\right)$ of the loss function:

(22) $$\phi \left(r\right)=\frac{d\rho \left(r\right)}{dr}$$

A common Huber weighting function is described as

(23) $$\varphi \left(r\right)=\left\{\begin{array}{cc} 1, & r\leq \delta ,\\ \frac{\delta }{\left|r\right|}, & r>\delta \end{array}\right.$$

This function assigns a lower weight to individual residuals with large normalized magnitudes. By combining the global weight decay factor $\alpha_{k}$ and the weight function estimated by M, the final weights can be expressed as follows:

(24) $$\begin{array}{cc} \omega_{GNSS,i} & =\alpha_{l}\cdot \varphi \left(\frac{r_{GNSS,i}}{\sigma_{GNSS}}\right)\\ \omega_{IMU,i} & =\alpha_{l}\cdot \varphi \left(\frac{r_{IMU,j}}{\sigma_{IMU}}\right)\\ \omega_{vision,i} & =\alpha_{l}\cdot \varphi \left(\frac{r_{vision,i}}{\sigma_{vision}}\right) \end{array}$$

The weights calculated in Equations (17)–(19) are not applied to the final state estimates directly. Instead, they are injected into the factor graph optimization framework defined in Equation (1) by dynamically scaling the information matrix of each sensor factor. In Equation (1), the term $∥\varphi \left(Z_{i},X\right){∥}_{P_{i}}^{2}$ represents the Mahalanobis distance $r^{T}\Lambda r$, where $\Lambda =P_{i}^{-1}$ is the information matrix. To implement adaptive weighting, the information matrix for sensor *l* is scaled by the global weight attenuation factor $\alpha_{l}$ and the M-estimation weight φ(·):

(25) $$\Lambda_{l}^{\mathrm{adj}}=\alpha_{l}\cdot \varphi \left(\frac{r_{l,i}}{\sigma_{l}}\right)\cdot \Lambda_{l}^{0}$$

where $\Lambda_{l}^{0}$ is the initial information matrix and l∈{GNSS,IMU,Vision}. Consequently, the optimization problem becomes

(26) $$X^{*}=\underset{X}{\mathrm{argmin}}\left\{∥\varphi_{p}-H_{p}{X∥}^{2}+\sum_{l}{\left(r_{l}\left(X\right)\right)}^{T}\Lambda_{l}^{\mathrm{adj}}r_{l}\left(X\right)\right\}$$

By scaling $\Lambda_{l}^{\mathrm{adj}}$, the proposed method reduces the influence of faulty sensors on the state estimation $X^{*}$ (which contains p,v) without resorting to loosely coupled linear blending. The final position and velocity are extracted from $X^{*}$ after solving the nonlinear least-squares problem.

**Figure 3:**
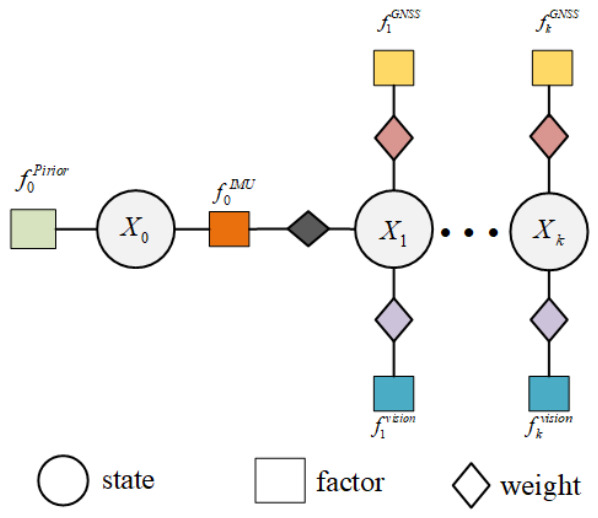
Factor graph model [1], where the boxes represent the a priori information, GNSS, IMU and visual sensor factors, the circles represent the system state at different moments, and the diamonds represent the weight factors of the three sensors.

In this way, the system can adaptively adjust the weights of different sensor types according to the dynamic changes in the residuals of the sensor observations and ensure that the effects of outliers is minimized while improving the robustness of the system. The specific optimization model is as follows: As shown in the Figure 3, a dynamic weighting factor is introduced to directly include or down-weight the sensor observations. This reduces the effects of sensor measurement errors on the positioning accuracy in complex environments. The specific Algorithm 1 is as follows:
**Algorithm 1** Adaptive weighting algorithm**Require:**
 Residuals $r_{\rho }^{\left(k\right)},r_{IMU}^{\left(k\right)},r_{vision}^{\left(k\right)}$**Ensure:**
 Optimized state $X^{*}$ (positions *p*, velocities *v*)1:Normalize residuals and compute test statistic $S_{l}$ via (16)2:Set $\Lambda_{l}=\Lambda_{l}^{0}$;    $\alpha_{l}\leftarrow \left\{\begin{array}{cc} 1, & S_{l}\leq T_{l}\\ T_{l}/S_{l}, & S_{l}>T_{l} \end{array}\right.$;   $\Lambda_{l}\leftarrow \alpha_{l}\Lambda_{l}$3:Apply M-estimator weights $w_{l,i}=\varphi \left(r_{l,i}/\sigma_{l}\right)$ to all residuals4:Build factor graph with updated $\Lambda_{l},w_{l,i}$ and solve (1) via Ceres to obtain $X^{*}$5:Extract p,v from $X^{*}$ and return

## 4. Real-World Dataset Evaluation and Analysis

In this section, the proposed adaptive weighted factor graph-optimized positioning algorithm based on joint GNSS/IMU/vision residual detection is comprehensively validated in different scenarios, including complex open-sky and indoor–outdoor environments, through offline experimental validation with public datasets. All evaluation metrics, including Root Mean Square Error (RMSE in meters) and Absolute Trajectory Error (ATE in meters), are clearly defined with their units and calculated over the entire trajectory unless otherwise specified.

### 4.1. Open Sky

This test was validated using sports field sequences from the GVINS dataset [1], which were collected in an open-air sports field using a helmet with a VI sensor and a u-blox ZED-F9 P. The GNSS RTK solution was used as the ground-truth solution. According to Cao et al, a fixed solution of the GNSS RTK was used as the ground truth. As no reference pose was provided, this experiment focused on evaluating the localization performance. The proposed system was compared with several FGO-based GNSS visual inertial fusion methods, including GVINS and VINS-Mono [26]. The GVINS system itself is a tightly coupled FGO-based fusion algorithm that also uses a sliding window for optimization.

**Figure 4:**
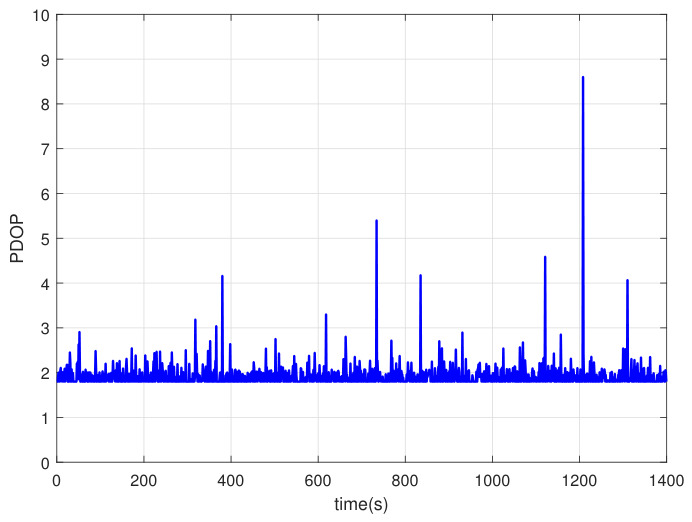
PDOP.

Figure 4 illustrates the GNSS observation conditions within the experimental environment in which the observability of the system was assessed using the PDOP. Throughout the experiment, the majority of satellites could be stably tracked, with PDOP values consistently remaining at approximately two. This indicates favorable observation conditions and an optimal configuration of the satellite geometry, thus supporting the reliability and accuracy of GNSS-based navigation methods.

**Figure 5:**
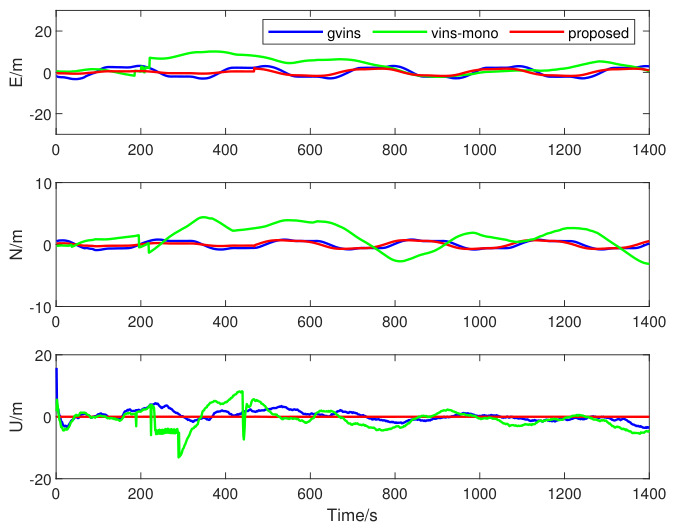
Localization errors of GVINS, VINS-Mono and the proposed algorithm in the open-sky dataset.

Figure 5 shows the positioning errors associated with the three positioning methods. As depicted in the figure, VINS-Mono exhibits significant positional deviations in all directions, which are primarily due to the accumulation of sensor errors. Similarly, GVINS exhibits a notable deviation in the easterly direction, which is attributed to the uncorrected GNSS error terms. In contrast, the proposed algorithm effectively solves these problems by integrating error detection and weight-adjustment techniques. Consequently, it achieves more consistent and accurate positioning results in all directions.

### 4.2. Complex Indoor–Outdoor Environments

For the validation test, complex indoor and outdoor environment sequences from the GVINS dataset were used to evaluate the robustness of the proposed system. The experimental trajectories encompass various challenging scenarios that single-sensor localization systems usually cannot cope with. For example, extreme illumination changes can impair the effective detection of feature points, whereas indoor environments or in areas with strong signal interference, the GNSS becomes almost unusable. In addition, the trajectory resembles common exploration tasks in that it does not have a distinct closed-loop structure, which further complicates the ability of visual–inertial systems to fully mitigate drift-related problems.

**Figure 6:**
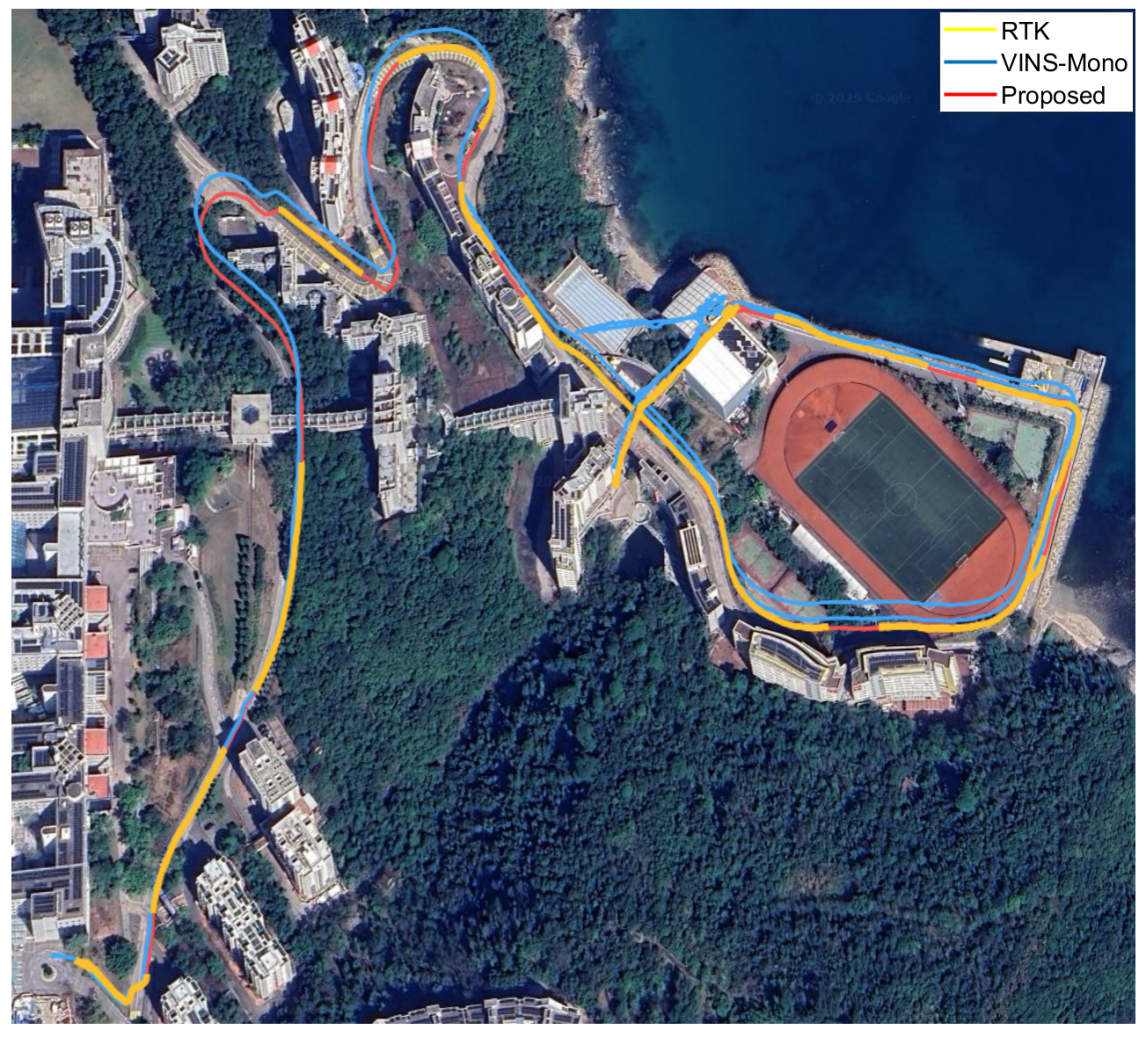
Final trajectories of complex indoor–outdoor experiments.

**Figure 7:**
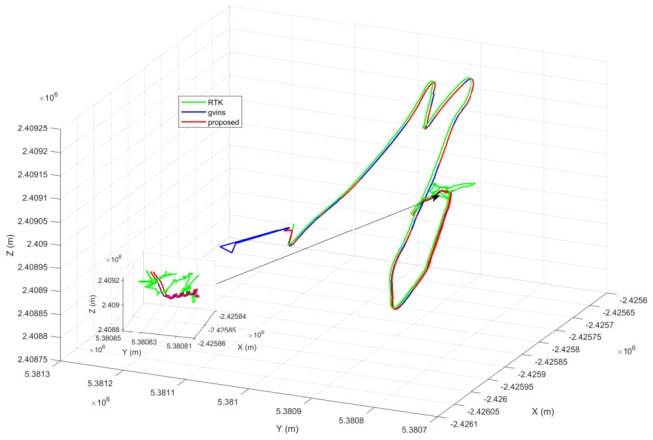
3D localization trajectory.

Figure 6 illustrates the final localization trajectory within a complex environment, and the corresponding 3D localization trajectory is presented in Figure 7. It can be seen that the trajectory generated by the proposed algorithm (shown in red) closely aligns with the RTK ground truth trajectory (shown in yellow) for most of the path, highlighting its exceptional spatial localization accuracy. However, in the indoor staircase area, as magnified in the figure, the RTK trajectory exhibits a significant deviation due to GNSS signal blockage. In addition, the GVINS trajectory shows a noticeable drift towards the end of the localization path, which was caused by error accumulation. In contrast, the proposed algorithm effectively tracks the real path in 3D space by adaptively weighting the fusion of the GNSS, IMU, and vision sensor data. These results emphasize the capability of the algorithm for accurate 3D trajectory tracking and its superior performance in high-precision navigation tasks.

The following section provides a detailed analysis of the localization performance of different algorithms to further examine the error characteristics of the proposed algorithm and their effects. Special attention is given to complex environments, where observing error variations in different directions provides valuable insights into the anti-interference capabilities and overall robustness of the algorithms.

**Figure 8:**
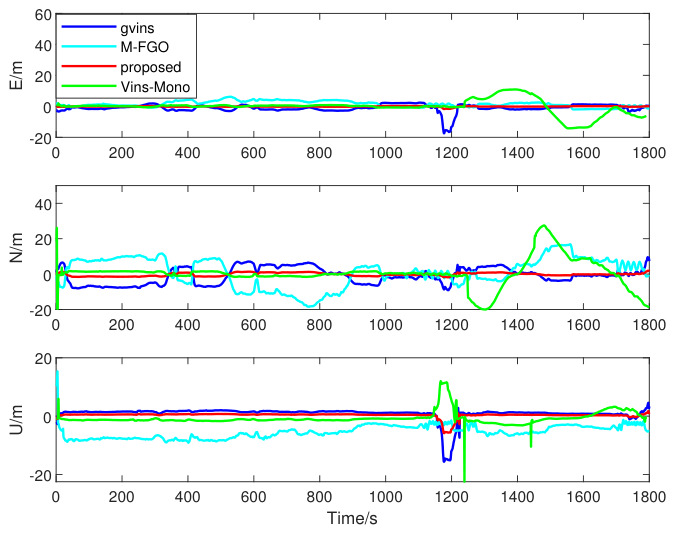
Positioning error.

From the positioning error curves in the East, North, and Up directions shown in Figure 8, it can be observed that throughout the entire evaluation period, the error of the proposed algorithm (red curve) is significantly lower than that of GVINS (blue curve) and VINS-Mono (green curve). It also demonstrates superior error suppression capability compared to the factor graph optimization method that uses only robust M-estimation, M-FGO (cyan curve). Particularly during the strong interference interval from 1200 s to 1400 s, the positioning error of GVINS increases sharply. While M-FGO manages to partially suppress error growth, it still exhibits noticeable fluctuations. In contrast, by incorporating a robust M-estimation mechanism into the factor graph optimization framework to dynamically adjust observation weights, the proposed algorithm effectively curbs the spread of errors and maintains a more stable error level across all three directions. Table 1 summarizes the RMSE and yaw error comparisons among the algorithms within the experimental environment. Overall, the proposed algorithm achieves superior error control, maintaining stable positioning accuracy over varying time periods and demonstrating enhanced robustness and precision.

**Figure 9:**
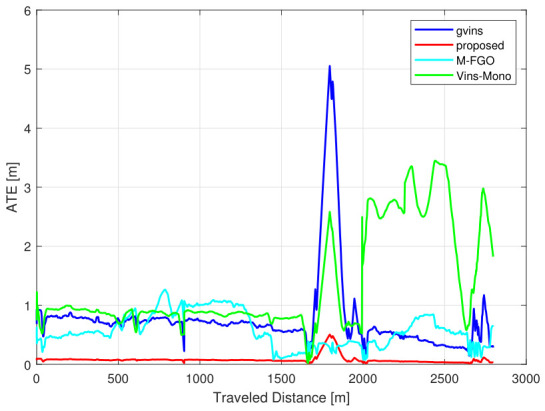
Absolute trajectory error.

Figure 9 illustrates the Absolute Trajectory Error curves for different algorithms over the traveled distance. Throughout the entire trajectory of 0–3000 m, the proposed algorithm (red curve) consistently maintains the lowest ATE and demonstrates the most stable error profile, reflecting its superior overall positioning accuracy and stability. Notably, around the 2000 m mark, where GNSS signals are likely subjected to interference, the GVINS method (blue curve) exhibits a significant error spike. In contrast, the proposed algorithm effectively suppresses the error surge through its robust fusion mechanism, maintaining smooth and low-error output. Meanwhile, although the M-FGO (cyan curve) and VINS-Mono (green curve) do not show drastic fluctuations, their overall error levels remain higher than that of the proposed algorithm. These results further validate the comprehensive advantage of the proposed algorithm in sustaining high precision and robustness over long-distance and complex environmental conditions.

**Figure 10:**
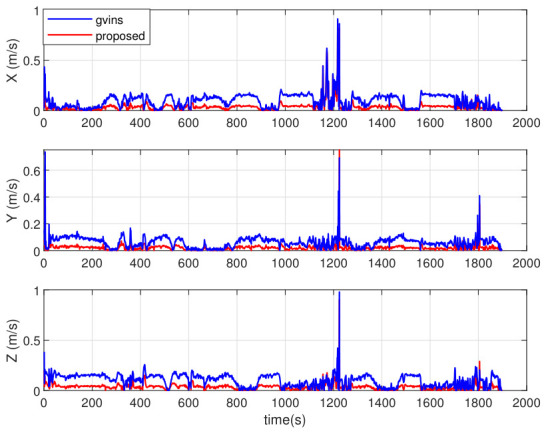
Velocity error.

To comprehensively evaluate the performance of the algorithms in dynamic environments, their accuracy and stability in velocity estimation are analyzed. Figure 10 illustrates the velocity errors of the proposed algorithm and GVINS in the X, Y, and Z directions. The key contribution of this figure is validating the effectiveness of the proposed adaptive weighting mechanism in suppressing velocity error accumulation. Specifically, the proposed algorithm maintains its velocity error within 0.25 m/s for most of the evaluation period. In contrast, GVINS exhibits significant fluctuations at approximately 1200 s due to GNSS signal interference, with peak errors reaching nearly 0.9 m/s. These results demonstrate that by dynamically adjusting sensor weights, the proposed algorithm effectively mitigates velocity error accumulation in dynamic environments, significantly enhancing the stability and anti-interference capability of velocity estimation.

In contrast, the proposed algorithm consistently maintains a low error level throughout the evaluation period. This performance advantage is attributed to the adaptive weighting mechanism integrated into the proposed algorithm, which is particularly effective in velocity estimation. By dynamically adjusting the sensor data weights in response to environmental changes or sensor fluctuations, the algorithm effectively suppresses the accumulation of velocity errors. As a result, the velocity estimation achieved by the proposed algorithm is not only smoother but also more accurate, demonstrating its robustness and adaptability in dynamic and challenging real-world environments.

## 5. Conclusions

In this study, an adaptive weighted factor graph optimization algorithm that incorporates joint residual detection was presented. The algorithm accumulates residual information from the GNSS pseudo-range, IMU pre-integration, and visual sensors within a sliding window framework and uses a combination of global weighting attenuation factors and the M-estimation method. This approach allows dynamic adjustment of the weights assigned to the different sensor data types in real time, effectively mitigating the effects of outliers on the state estimation. Offline experimental validation with real-world datasets shows that the proposed algorithm achieves superior positioning accuracy and robustness in both open-sky conditions and complex indoor–outdoor environments. In particular, the algorithm significantly reduces error accumulation and maintains a smooth 3D trajectory and velocity estimation, even under conditions of GNSS signal interference or blockage. This demonstrates a strong anti-interference capability, adaptability and versatility, making it well-suited for applications in autonomous driving and urban IoT. Future work will focus on dynamic modeling and compensation of sensor errors to further improve the positioning accuracy and adaptability of the system to diverse environmental conditions.

## Figures and Tables

**Table 1:** Comparison of RMSE and yaw errors.

	Proposed	GVINS	VINS-Mono	M-FGO
RMSE (m)	2.505	3.700	6.905	4.713
Yaw error (deg)	2.010	2.684	4.935	2.985

## Data Availability

The data presented in this study are available on request from the corresponding author. The data are not publicly available due to privacy and commercial confidentiality restrictions related to the sensor hardware and experimental platform.

## Abbreviations

The following abbreviations are used in this manuscript:

GNSS	global navigation satellite systems
GVINS	GNSS–Visual–Inertial Navigation System
IMU	inertial measurement units
FGO	factor graph optimization
INS	inertial navigation systems
EKF	extended Kalman filter
6-DOF	6-degree-of-freedom
BLUE	Best Linear Unbiased Estimator

## References

[1] Cao S., Lu X., Shen S. (2022). GVINS: Tightly Coupled GNSS-Visual-Inertial Fusion for Smooth and Consistent State Estimation. IEEE Trans. Robot..

[2] Li T., Zhang H., Gao Z., Niu X., El-sheimy N. (2019). Tight Fusion of a Monocular Camera, MEMS-IMU, and Single-Frequency Multi-GNSS RTK for Precise Navigation in GNSS-Challenged Environments. Remote Sens..

[3] Qin T., Cao S., Pan J., Shen S. (2019). A General Optimization-based Framework for Global Pose Estimation with Multiple Sensors. arXiv.

[4] Li X., Xia C., Li S., Zhou Y., Shen Z., Qin Z. (2023). A Filter-Based Integration of GNSS, INS, and Stereo Vision in Tight Mode with Optimal Smoothing. IEEE Sens. J..

[5] Angelino C.V., Baraniello V.R., Cicala L. UAV position and attitude estimation using IMU, GNSS and camera. Proceedings of the 15th International Conference on Information Fusion.

[6] Shen S., Mulgaonkar Y., Michael N., Kumar V. Multi-sensor fusion for robust autonomous flight in indoor and outdoor environments with a rotorcraft MAV. Proceedings of the IEEE International Conference on Robotics and Automation (ICRA).

[7] Sun R., Yang Y., Chiang K.-W., Duong T.-T., Lin K.-Y., Tsai G.-J. (2020). Robust IMU/GPS/VO Integration for Vehicle Navigation in GNSS Degraded Urban Areas. IEEE Sens. J..

[8] Indelman V., Williams S., Kaess M., Dellaert F. (2013). Information fusion in navigation systems via factor graph based incremental smoothing. Robot. Auton. Syst..

[9] Cheng X., Peng X., Wu D., Zhang X. Multi-Sensor Fusion System Based on Factor Graph Optimization. Proceedings of the 2023 42nd Chinese Control Conference (CCC).

[10] Bai S., Lai J., Lyu P., Ji B., Wang B., Sun X. (2023). A Novel Plug-and-Play Factor Graph Method for Asynchronous Absolute/Relative Measurements Fusion in Multisensor Positioning. IEEE Trans. Ind. Electron..

[11] Liu X., Wen S., Jiang Z., Tian W., Qiu T.Z., Othman K.M. (2023). A Multisensor Fusion with Automatic Vision–LiDAR Calibration Based on Factor Graph Joint Optimization for SLAM. IEEE Trans. Instrum. Meas..

[12] Ma T., Chen S., Ruan L., Xu Y. A Vision-Integrated Navigation Method in AUV Terminal Mobile Docking Based on Factor Graph Optimization. Proceedings of the 2023 8th International Conference on Automation, Control and Robotics Engineering (CACRE).

[13] Mascaro R., Teixeira L., Hinzmann T., Siegwart R., Chli M. GOMSF: Graph-Optimization Based Multi-Sensor Fusion for robust UAV Pose estimation. Proceedings of the 2018 IEEE International Conference on Robotics and Automation (ICRA).

[14] Li X., Chang H., Wang X., Li S., Zhou Y., Yu H. (2024). An Optimization-Based Tightly-Coupled Integration of PPP, INS and Vision for Precise and Continuous Navigation. IEEE Trans. Veh. Technol..

[15] Niu X., Tang H., Zhang T., Fan J., Liu J. (2023). IC-GVINS: A Robust, Real-Time, INS-Centric GNSS-Visual-Inertial Navigation System. IEEE Robot. Autom. Lett..

[16] Dong X., Hu G., Gao B., Zhong Y., Ruan W. (2024). Windowing-Based Factor Graph Optimization with Anomaly Detection Using Mahalanobis Distance for Underwater INS/DVL/USBL Integration. IEEE Trans. Instrum. Meas..

[17] Ben Y., Wang K., Li Q. (2024). A Robust Factor Graph Optimization Method for Navigation in Land Vehicles Based on Dynamic Kernel Principal Component Analysis. IEEE Trans. Instrum. Meas..

[18] Zhou Y., Chen X., Ge M. Robust Factor Graph Optimization Integrated Navigation Based on Improved Chi-Square Test. Proceedings of the 2023 International Conference on Sensing, Measurement & Data Analytics in the era of Artificial Intelligence (ICSMD).

[19] Wei X., Li J., Zhang D., Feng K. (2021). An improved integrated navigation method with enhanced robustness based on factor graph. Mech. Syst. Signal Process..

[20] Fan G., Wang Q., Yang G., Liu P. (2024). RFG-TVIU: Robust factor graph for tightly coupled vision/IMU/UWB integration. Front. Neurorobot..

[21] Gao L., Xiang H., Xia X., Ma J. (2024). Multisensor Fusion for Vehicle-to-Vehicle Cooperative Localization With Object Detection and Point Cloud Matching. IEEE Sens. J..

[22] Liu B., Liu G., Wei S., Su G., Wang J. On Improved Algorithm of SINS/DR Integrated Navigation System. Proceedings of the 2018 IEEE CSAA Guidance, Navigation and Control Conference (CGNCC).

[23] Sun R., Dai Y., Cheng Q. (2023). An Adaptive Weighting Strategy for Multisensor Integrated Navigation in Urban Areas. IEEE Internet Things J..

[24] Jung M., Jung S., Kim A. (2023). Asynchronous Multiple LiDAR-Inertial Odometry Using Point-Wise Inter-LiDAR Uncertainty Propagation. IEEE Robot. Autom. Lett..

[25] Dellaert F., Kaess M. (2017). Factor Graphs for Robot Perception. Found. Trends Robot..

[26] Qin T., Li P., Shen S. (2018). VINS-Mono: A robust and versatile monocular visual-inertial state estimator. IEEE Trans. Robot..

